# Effectiveness of SGLT2 Inhibitors in Type 2 Diabetes: A Systematic Integrative Review of Reviews and Comparative Effectiveness Studies (2020–2025)

**DOI:** 10.3390/pharmacy14020047

**Published:** 2026-03-12

**Authors:** Desislava Stanimirova, Guenka Petrova, Zornitsa Mitkova

**Affiliations:** 1 Faculty of Pharmacy, Department of Organisation and Economy of Pharmacy, Medical University Sofia, 2 Dunav Street, 1431 Sofia, Bulgaria; 2 Research Institute of Innovative Medical Science, Medical University Sofia, 2 Dunav Street, 1431 Sofia, Bulgaria

**Keywords:** SGLT2 inhibitors, diabetes, systematic integrative review, cardiovascular outcomes, renal outcomes

## Abstract

This systematic integrative review evaluates the effectiveness of SGLT2 inhibitors in relation to improving glycaemic control, reducing cardiovascular events, and preserving renal function based on the latest published evidence. Search for publications referenced in PubMed, from January 2020 to January 2025, was conducted; 48 abstracts were reviewed, and 27 full-text articles were included for analysis—systematic reviews, meta-analyses, narrative reviews and comparative effectiveness studies. SGLT2 inhibitors are effective in reducing glucose levels, but the magnitude of reduction varies compared to other classes of antidiabetics. A noticeable reduction in the risk of major cardiovascular events, cardiovascular and all-cause mortality was reported, particularly compared to DPP-4 inhibitors and placebo. SGLT2 inhibitors demonstrated the most pronounced and consistent benefits in reducing hospitalisation for heart failure among all other evaluated classes. However, outcomes like myocardial infarction and stroke results were inconsistent. Renal outcomes consistently favoured SGLT2 inhibitors in reducing the risk of acute kidney injury, slowing chronic kidney disease and lowering the risk of end-stage kidney disease. SGLT2 inhibitors provide consistent glucose-lowering, cardiovascular and renal benefits. However, heterogeneity in study designs, patient populations, and treatment durations does not allow drawing definitive conclusions and highlights the need for future research focused on conducting well-designed trials with standardised methodology.

## 1. Introduction

Type 2 diabetes mellitus (T2DM) is a chronic metabolic disorder, which is characterised by insulin resistance, pancreatic beta-cell dysfunction and subsequent hyperglycaemia, affecting millions of people worldwide [1]. This disease is associated with many serious complications, such as cardiovascular disease (CVD), chronic kidney disease (CKD) and neuropathy. Those complications contribute to increased morbidity and mortality [2].

Approximately 537 million adults worldwide have diabetes. This makes the disease one of the fastest-growing burdens of the present time. It is expected that by 2045, more than 738 million people will be diabetic [3]. In 2021, diabetes global health expenditures were estimated at 966 billion USD—a number that is expected to rise up to 1054 billion USD by 2030 [4]. Timely diagnoses and access to treatment are essential for enhancing diabetes outcomes; their absence exacerbates the disease burden among impoverished populations. This emphasises the importance of global health policies regarding lifestyle interventions, preventive measures and access to treatment.

Treatment of T2DM consists of lifestyle modifications, oral antidiabetic drugs, and, in some cases, insulin therapy. Although the complex treatment assists in keeping diabetes under control, there are still limitations that need to be conquered—weight gain, risks of hypoglycaemia, and insufficient protection against cardiovascular and renal complications. This brought the need for the creation of new classes of antidiabetic medications that can affect glucose levels while simultaneously possessing additional metabolic and cardiovascular benefits.

Sodium–glucose co-transporter 2 (SGLT2) inhibitors with main agents—empagliflozin, dapagliflozin, and canagliflozin—have emerged as a transformative class of antidiabetic medications, which offer benefits beyond glucose control [5]. SGLT2 inhibitors affect renal glucose reabsorption by blocking the SGLT2 co-transporters in the proximal tubules. That action causes glucosuria, followed by a reduction in glucose levels and HbA1c values. The additional excretion of sodium reverts the tubuloglomerular feedback and reduces intraglomerular pressure, which is a key component of the nephroprotective effects of SGLT2 inhibitors. Losing calories improves lipid metabolism and insulin sensitivity—this shift in metabolism toward gluconeogenesis and ketogenesis protects the cardiovascular and renal systems [6].

SGLT2 inhibitors have been thoroughly studied for their cardiovascular benefits, including reduced MACE and heart failure hospitalisation. Results have been shown in line with large clinical trials such as EMPA-REG OUTCOME, CANVAS, and DECLARE-TIMI 58 [7]. Other large clinical trials—CREDENCE, DAPA-CKD, and EMPA-KIDNEY—focused on the protective function of SGLT2 inhibitors on the renal system, with results showing that medication from the class slows down the progression of CKD and albuminuria [8].

Due to the wide-ranging impact on different organ systems, SGLT2 inhibitors have gained recognition. Despite the benefits, challenges—such as concerns of long-term safety, costs, and potential side effects like diabetic ketoacidosis and genitourinary infections—still remain [9].

The aim of this systematic review is to evaluate the effectiveness of SGLT2 inhibitors related to improvement of glycaemic control, reduction in cardiovascular events, and preservation of renal function based on the latest published evidence. Unlike previous reviews focusing on single outcome or study design, this review integrates mixed levels of evidence—systematic reviews, meta-analyses, narrative reviews, as well as comparative effectiveness studies within a single framework and aims to evaluate not a single but multiple different outcomes. This allows us to make a direct comparison of findings across real-world studies and randomised trials and to better understand the consistency and variability of treatment effects.

## 2. Materials and Methods

### 2.1. Study Design

A systematic integrative review of the published literature that evaluates the effectiveness of SGLT2 inhibitors in patients with type 2 diabetes was performed. It was designed as a systematic integrative review, which includes mixed levels of evidence: both primary evidence—comparative effectiveness studies—and secondary evidence—systematic review, meta-analyses and narrative reviews. This helped to provide a broad and comprehensive overview of both clinical and real-world effectiveness. Our objective was to synthesise trends across different types of evidence. This review follows the Preferred Reporting Items for Systematic Reviews and Meta-Analyses (PRISMA) guidelines to ensure a structured, transparent, and reproducible approach to data collection and synthesis. A completed PRISMA checklist is provided in the Supplementary Materials (File S1) [10]. The primary objective of the review is to assess the impact of SGLT2 inhibitors on glycaemic control, cardiovascular outcomes, and renal function in patients with type 2 diabetes. This systematic integrative review was registered in PROSPERO (CRD420261287061), where the protocol is publicly available.

### 2.2. Literature Search Strategy

For the identification of studies, a comprehensive literature search in the electronic database PubMed was performed. This database was selected as the primary source of information because of its extensive indexing of peer-reviewed biomedical and clinical literature. Additional databases were not searched, which may have limited the retrieval of some relevant studies. The search covered all studies published between the years 2020 and 2025. The keywords that were used included the following: “SGLT2 inhibitors” AND “type 2 diabetes” AND “effectiveness”. All relevant titles and abstracts were screened for relevance, and selected full-text articles were retrieved for a detailed review.

### 2.3. Inclusion and Exclusion Criteria

Specific inclusion and exclusion criteria were determined. All included studies needed to meet the following criteria: systematic reviews, meta-analyses, narrative reviews or comparative effectiveness studies, observational and retrospective cohort studies published between 2020 and 2025, evaluating the impact of SGLT2 inhibitors on glycaemic control, cardiovascular outcomes, and renal function in patients with type 2 diabetes, and written in the English language. Articles were included if they provided information regarding diabetes patients on at least one of the three primary endpoints: glycaemic control—HbA1c or fasting plasma glucose reduction; cardiovascular outcomes—major adverse cardiovascular event, heart failure hospitalisation, and mortality; or renal function—progression of CKD, AKI, and reaching the end-stage kidney disease. The criteria for exclusion were non-human subjects, case reports, articles that lack information about patients with type 2 diabetes, articles published before 2020, and articles focused on safety without reporting effectiveness. The list of excluded studies is provided in the Supplementary Materials (Table S1).

### 2.4. Study Selection

Titles and abstracts were initially screened to assess their relevance; full-text articles were reviewed based on the inclusion and exclusion criteria. The abstracts and titles of the identified sources were screened independently by two authors. In case of disagreement, the third one served as the “referee”. The selection process adhered to PRISMA guidelines to ensure a comprehensive and unbiased approach [10].

### 2.5. Data Extraction

The data extraction was performed by one author. All of the extracted information was cross-checked for accuracy and consistency. It was then independently verified by the other two authors. Discrepancies were resolved by consensus. The data extraction was performed systematically using the following details from each article (Table 1): first author and year of publication, type of evidence, study design, sample size, as well as availability of outcomes related to glucose level control, cardiovascular events, and renal function. In addition, the overall assessment and the tools used for it were recorded.

For the comparative effectiveness studies, detailed data was collected, including the number of patients, mean age, study region, duration of exposure and follow-up, specific SGLT2 inhibitors, if they were pinpointed, comparators and reported cardiovascular and renal outcomes with their hazard ratios or relative risks with corresponding 95% confidence intervals, when available. The summarised information is available in Table 2.

For the systematic reviews, meta-analyses and narrative reviews, extracted data included study types, numbers of studies included, mean age, evaluated SGLT2 inhibitors and comparator drug classes, main cardiovascular and renal findings of the studies and pooled effect estimates, where they were provided.

The extracted data was used for a structured narrative synthesis comparing the effectiveness of SGLT2 inhibitors.

### 2.6. Data Analysis

The effectiveness of the SGLT2 inhibitors was assessed based on reported changes in HbA1c levels, cardiovascular risk reduction, and renal protection. A narrative synthesis was conducted in order to summarise trends and to draw a general conclusion about the key findings. A quantitative meta-analysis was not performed due to the heterogeneity between the included studies in terms of study design, populations, comparators and reported outcomes.

### 2.7. Assessment of the Risk of Bias

For observational studies, ROBINS-I was used to assess risk of bias, evaluating bias resulting from confounding, selection of participants, classification of interventions, deviations from intended interventions, missing data, measurement of outcome and selective reporting [11]. AMSTAR 2 was used to evaluate overall methodological confidence for systematic reviews and meta-analyses, across domains including protocol registration, literature search adequacy, risk-of-bias assessment, synthesis methods and interpretation of results [12]. Narrative reviews were assessed with the SANRA checklist for reporting quality [13] and were not evaluated for risk of bias. Each study was determined to have low, moderate, or serious risk of bias (for observational studies) and with high, moderate or critically low methodological confidence (for systematic review and meta-analyses). Assessment of the risk of bias, methodological confidence and reporting quality was performed independently by two authors. In case of disagreement, the third one served as the “referee”.

## 3. Results

### 3.1. Search Results

**Figure 1:**
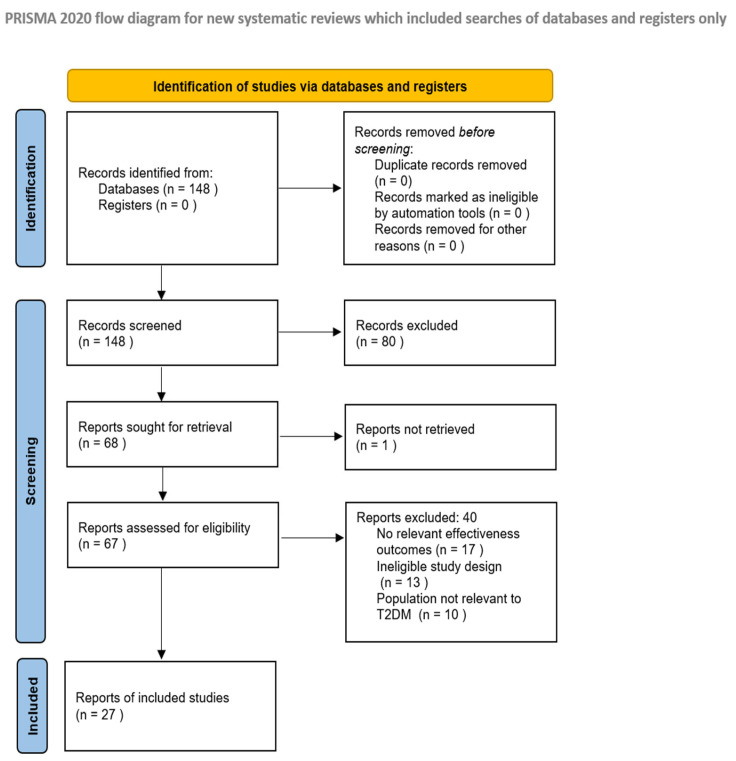
PRISMA diagram, 2020.

The literature search was performed exclusively in the electronic database PubMed. PubMed was selected as a database systematising most medical articles. A total of 148 relevant articles containing systematic reviews, meta-analyses, and comparative effectiveness studies were identified. No duplicate records were identified during screening or excluded by automation tools or for other reasons prior to the screening. After the initial screening of the titles and abstracts, 80 articles were excluded. A total of 68 reports were sought for full-text retrieval, as one report could not be retrieved. A total of 67 full-text reports were assessed for eligibility. After full-text screening, 40 more articles were excluded. The total number of articles included was 27 (Figure 1). Risk-of-bias and methodological quality assessment were conducted after the study selection. Observational comparative effectiveness studies were mainly assessed as having moderate risk of bias (nine studies), low risk of bias (three studies), and serious risk of bias (two studies) using ROBINS-I. Systematic reviews and meta-analyses evaluated with AMSTAR 2 demonstrated again mainly moderate confidence (5 studies), high confidence (2 studies), and 3 studies showed critically low confidence. All three narrative reviews showed adequate reporting quality according to SANRA and were used to support qualitative interpretations of findings (Table 1). Following an initial screening of titles and abstracts, studies were evaluated based on predefined eligibility criteria. After full-text screening, systematic reviews, meta-analyses and comparative effectiveness studies were included if they provided data on at least one of the three primary endpoints: glycaemic control (HbA1c), cardiovascular outcomes (major adverse cardiovascular events, heart failure hospitalisation, and mortality), or renal function (progression of chronic kidney disease, acute kidney injury, end-stage kidney disease and composite renal outcome/renal events).

### 3.2. Characteristics of Included Studies

The studies included in this review varied in design, sample size, and population characteristics. They were published in the period from January 2020 to January 2025 (Table 1). The number of analysed studies in each article differed as well—a combination of randomised controlled trials (RCTs) and observational cohort studies was included. The range of sample size varied from 5005 to 1,300,184 patients, covering various subgroups, including patients with type 2 diabetes and high renal or cardiovascular risk. Comparators used in the studies included placebo, dipeptidyl peptidase 4 inhibitors (DPP4 inhibitors), glucagon-like peptide-1 receptor agonists (GLP1 RA), and other glucose-lowering drugs (oGLDs), insulin, metformin, sulfonylureas and other standard therapy or combination of therapies for diabetes. The primary endpoints of interest were glycaemic control, cardiovascular outcomes, and renal function, with data extracted and summarised from multiple systematic reviews, meta-analyses, and comparative effectiveness studies (Table 2 and Table 3).

### 3.3. Effect on Glycaemic Control

HbA1c is the parameter used to observe the sustained control of glucose levels. SGLT2 inhibitors have demonstrated a modest but consistent impact on glycaemic control. Despite the fact that some studies lack clear quantitative measures, others report improvement in glycaemic control between 0.85% to 1.0% [14,15]. Compared to DPP4 inhibitors, SGLT2 inhibitors show a slight advantage, particularly in younger patients, lowering the HbA1c levels with 0.4% more [16]. Real-world data further confirm the superiority of SGLT2 inhibitors, with 3.3% more patients reaching HbA1c levels below 7.0% compared to DPP4 inhibitors [17]. Improvement of glycaemic control was noted in broad patient populations, but the magnitude of reduction varied.

**Table 1:** Included studies.

Authors, Year of Publication	Type of Evidence	Study Design	Study Population	Glycaemic Control	Cardiovascular Events	Renal Events	Overall Assessment	Tool Used *
D’Andrea et al., 2023 [18]	Comparative Effectiveness Study	Observational Cohort Study	144,614	-	Reported	Reported	Moderate risk of bias	ROBINS-I
Drake et al., 2024 [19]	Systematic Review and Network Meta-analysis	84 RCT	not stated	-	Reported	Reported	High confidence	AMSTAR 2
Edmonston et al., 2024 [20]	Comparative Effectiveness Study	Observational Cohort Study	82,272	-	Reported	Reported	Moderate risk of bias	ROBINS-I
Xie et al., 2023 [21]	Comparative Effectiveness Study	Observational Cohort Study	283,998	-	Reported	-	Low risk of bias **	ROBINS-I
Fadini et al., 2024 [22]	Comparative Effectiveness Study	Observational Cohort Study	12,394	Reported	-	Reported	Moderate risk of bias	ROBINS-I
Wheeler et al., 2020 [23]	Narrative Review	5 Major RCT and Observational Studies	not stated	-	Reported	Reported	High reporting quality	SANRA
Fralick et al., 2021 [24]	Comparative Effectiveness Study	Observational Cohort Study	19,928	-	Reported	Reported	Serious risk of bias	ROBINS-I
Sinha et al., 2024 [25]	Systematic Review and Meta-Analysis	Total of 12 studies—5 RCTs and 7 Observational studies	Not stated	-	Reported	-	Critically low confidence	AMSTAR 2
Singh et al., 2023 [14]	Systematic Review	RCTs and Observational Studies	Not stated	Reported	Reported	Reported	Critically low confidence	AMSTAR 2
Kalluri et al., 2021 [26]	Systematic Review	Total of 22—18 RCT and 4 Observational Studies	702,977	Reported	Reported	-	Critically low confidence	AMSTAR 2
Caparrotta et al., 2021 [27]	Systematic Review	37 Observational Studies	1,300,184	-	Reported	Reported	Moderate confidence	AMSTAR 2
Xie et al., 2020 [28]	Comparative Effectiveness Study	Observational Cohort Study	216,558	Reported	-	Reported	Low risk of bias **	ROBINS-I
Goh et al., 2022 [17]	Comparative Effectiveness Study	Observational Cohort Study	30,414	Reported	Reported	Reported	Moderate risk of bias	ROBINS-I
Dong et al., 2022 [29]	Comparative Effectiveness Study	Observational Cohort Study	26,032	-	Reported	-	Moderate risk of bias	ROBINS-I
Ueda et al., 2022 [30]	Comparative Effectiveness Study	Observational Cohort Study	151,446	-	Reported	Reported	Moderate risk of bias	ROBINS-I
Nyström, 2024 [31]	Narrative Review	RCTs and Observational Studies	Not stated	-	Reported	Reported	High reporting quality	SANRA
Maremmani et al., 2025 [32]	Systematic Review and Meta-Analysis	Total of 9—5 RCT and 4 Observational Studies	26,753	-	Reported	-	Moderate confidence	AMSTAR 2
Ahmed et al., 2024 [33]	Systematic Review and Meta-Analysis	5 RCT	11,211	-	Reported	-	High confidence	AMSTAR 2
Koh et al., 2021 [34]	Comparative Effectiveness Study	Observational Cohort Study	90,032	-	Reported	Reported	Moderate risk of bias	ROBINS-I
Cao et al., 2022 [35]	Systematic Review and Network Meta-Analysis	16 RCT	46,292	-	Reported	Reported	Moderate confidence	AMSTAR 2
Bonnet et al., 2024 [36]	Narrative Review	RCTs and Observational Studies	Not stated	Reported	Reported	Reported	High reporting quality	SANRA
Shiao et al., 2025 [37]	Comparative Effectiveness Study	Observational Cohort Study	5005	-	Reported	-	Serious risk of bias	ROBINS-I
Güdemann et al., 2024 [16]	Comparative Effectiveness Study	Observational Cohort Study	161,825	Reported	-	-	Moderate risk of bias	ROBINS-I
Han et al., 2021 [38]	Comparative Effectiveness Study	Observational Cohort Study	31,398	-	Reported	-	Moderate risk of bias	ROBINS-I
Martínez-Vizcaíno et al., 2021 [39]	Systematic Review and Network Meta-Analysis	6 RCT	46,969	-	Reported	Reported	Moderate confidence	AMSTAR 2
Xie et al., 2021 [40]	Comparative Effectiveness Study	Observational Cohort Study	128,293	-	Reported	-	Low risk of bias **	ROBINS-I
Teng et al., 2024 [15]	Systematic Review and Network Meta-Analysis	28 RCTs	8499	Reported	-	-	Moderate confidence	AMSTAR 2

* ROBINS-I ratings refer to risk of bias in observational studies; AMSTAR 2 ratings refer to methodological confidence of systematic reviews; SANRA ratings refer to reporting quality of narrative reviews and do not indicate risk of bias. ** A “low risk of bias” judgment under ROBINS-I reflects a liberal interpretation applied to target trial emulation studies and does not imply equivalence to randomised controlled trials.

**Table 2:** Summary of real-world comparative effectiveness studies evaluating SGLT2 inhibitors in type 2 diabetes.

RWE Studies	Patients, Number	Mean Age, Years	Regions/Countries	Duration	Follow-Up, Mean (Years)	SGLT2 Inhibitor	Comparator	Cardiovascular Outcome ^a^	Effect Measure (HR Unless Otherwise Specified) (95% CI)	Renal Outcome ^a^	Effect Measure (HR Unless Otherwise Specified) (95% CI)
D’Andrea et al., 2023 [18]	144,614	62	United States	2013–2021	0.66 y	Canagliflozin	DPP-4 inhibitors	MACE (modified)	0.85 (0.75–0.95)	AKI ^c^	0.64 (0.58–0.70)
						Dapagliflozin		HHF	0.46 (0.35–0.57)		
						Empagliflozin		MI	0.92 (0.74–1.09)		
						Ertugliflozin		Stroke	0.86 (0.65–1.08)		
								All-cause mortality	0.74 (0.59–0.88)		
Edmonston et al., 2024 [20]	82,272	59	United States	2015–2020	1.2 y	SGLT2 inhibitors (class)	GLP-1 RA	MACE	1.03 (95% CI 0.93–1.14)	40% eGFR decline	0.77 (0.65–0.91)
								HHF	0.95 (95% CI 0.80–1.13)		
								All-cause mortality	1.08 (95% CI 0.92–1.27)		
Xie et al., 2023 [21]	283,998	64.2	United States	Oct 2016–Sep 2021	3.85 y	Canagliflozin	DPP-4 inhibitors	MACE	0.86 (0.82–0.89)	NR	NR
						Dapagliflozin	GLP-1 RA	MACE	0.99 (0.94–1.04)		
						Empagliflozin	oGLD (SU)	MACE	0.77 (0.74–0.80)		
Fadini et al., 2024 [22]	12,394	61	Italy	Jan 2015–Sep 2020	2.5 y	Dapagliflozin	oGLDs (DPP-4 inhibitors (40%), GLP-1 RA (22.3%), sulfonylureas (16.1%), pioglitazone (8%), metformin (5.8%), acarbose (4%))	NR	NR	Composite kidney outcome	0.70 (0.56–0.87)
										New-onset CKD	0.76 (0.66–0.89)
										CKD progression	0.93 (0.87–0.99)
										≥40% eGFR decline	0.69 (0.56–0.87)
										≥57% eGFR decline	0.65 (0.44–0.96)
Fralick et al., 2021 [24]	19,928	54	United States	2013–2018	0.4–0.6 y	SGLT2 inhibitors (class)	oGLD (metformin)	Composite CV outcome (HF, MI, or stroke)	0.82 (0.58–1.15)	AKI ^c^	0.94 (0.60–1.47)
								HHF	0.81 (0.40–1.63)		
								MI	0.91 (0.54–1.52)		
								Stroke	0.76 (0.42–1.39)		
Xie et al., 2020 [28]	216,558	65.5	United States	Oct 2016–Nov 2019	1.6 y	SGLT2 inhibitor class	DPP-4 inhibitors	NR	NR	Composite kidney outcome	0.62 (0.51–0.75)
							GLP-1 RA			Composite kidney outcome	0.89 (0.68–1.15)
							oGLD (SU)			Composite kidney outcome	0.55 (0.46–0.67)
Goh et al., 2022 [17]	30,414	~57	Singapore (NHIS)	2015–2018	0.08–1 y	Dapagliflozin, Empagliflozin, Canagliflozin	DPP-4 inhibitors	HHF	0.78 (0.63–0.95) ^b^	Kidney complication	0.28 (0.20–0.39) ^b^
								All-cause mortality	0.66 (0.51–0.85) ^b^		
Dong et al., 2022 [29]	26,032	53	Taiwan (NHIRD)	2012–2018	0.6 y	Dapagliflozin, Empagliflozin	GLP-1 RA	MI	0.99 (0.65–1.52)	NR	NR
								Total Stroke	0.90 (0.69–1.17)		
								Ischenic Stroke	0.86 (0.65–1.14)		
								Haemorrhagic Stroke	0.88 (0.63–1.25)		
Ueda et al., 2022 [30]	151,446	61.4	Sweden, Denmark, Norway	2013–2018	1.6–2.2 y	SGLT2 inhibitors (class)	GLP-1 RA	MACE	1.07 (1.01–1.15)	Serious renal events	0.76 (0.66–0.87)
								HF (hospitalisation or death)	1.02 (0.92–1.12)		
								MI	1.09 (1.00–1.19)		
								Stroke	1.14 (1.03–1.26)		
								CV death	0.97 (0.84–1.12)		
								All-cause mortality	1.01 (0.94–1.09)		
Koh et al., 2021 [34]	90,032	58.1	South Korea (NHIS nationwide database)	2014–2017	1.49 y	Dapagliflozin, Empagliflozin, Ipragliflozin	oGLDs	All-cause mortality	0.82 (0.73–0.93)	ESRD	0.47 (0.34–0.65)
Shiao et al., 2025 [37]	5005	~60	Taiwan (NHIRD)	2016–2021	2.3 y	Dapagliflozin, Empagliflozin	DPP-4 inhibitors	MACE (4-point-MI, stroke, CV death, HHF)	0.72 (0.52, 1.00)	NR	NR
								CV death	0.37 (0.21, 0.65)		
							non-SGLT2i	MACE (4-point-MI, stroke, CV death, HHF)	0.68 (0.49, 0.95)		
								CV death	0.38 (0.21, 0.65)		
							oGLD (TZD)	MACE (4-point-MI, stroke, CV death, HHF)	0.56 (0.35, 0.88)		
								CV death	0.42 (0.20, 0.90)		
Han et al., 2021 [38]	31,398	71.9	South Korea (NHIS)	Sep 2014–Dec 2016	1.05 y	Dapagliflozin, Empagliflozin, Ipragliflozin	DPP-4 inhibitors	HHF	0.86 (0.76–0.97	NR	NR
								MI	0.95 (0.77–1.19)		
								Stroke	0.86 (0.77–0.97)		
								All-cause mortality	0.85 (0.75–0.98)		
								HHF + all-cause death (composite)	0.86 (0.78–0.94)		
Xie et al., 2021 [40]	128,293	64.6	United States	Oct 2016–Feb 2020	2.20 y	Canagliflozin, Dapagliflozin, Empagliflozin	oGLD (SU)	All-cause mortality	0.81 (0.75–0.87)	NR	NR

^a^—definitions of MACE, composite cardiovascular outcomes and composite renal outcomes varied by study and are reported as defined by the original authors. ^b^—effect estimates are pooled risk ratios (RRs); hazard ratios were not reported. ^c^—AKI represents short-term renal events rather than long-term renal effectiveness outcomes. NR—not reported.

### 3.4. Cardiovascular Outcomes

While conducting the review, it became clear that SGLT2 inhibitors contribute to reducing the risk of cardiovascular events across all different study designs. Across the included analyses [18,19,20,21,23,24,25,26,27,29,30,31,32,33,34,35,36,37,38,39,40], SGLT2 inhibitors show a consistent benefits, indicating a protective effect.

Systematic reviews and meta-analyses comparing SGLT2 inhibitors to mostly a placebo. SGLT2 inhibitors led to a lower risk of MACE events, with between 9% and 17%. When compared with DPP4 inhibitors, SLGT2 inhibitors demonstrated from 12% to 19% lower risk of MACE. SGLT2 inhibitors showed a neutral pooled effect with relative risk reductions with 5%, which was not a statistically significant result compared to GLP1 RA. Across the systematic reviews and meta-analyses, the reduction in hospitalisation for heart failure was the most consistent cardiovascular benefit. Compared to placebo, SGLT2 inhibitors showed a reduction from 14% to 30%. Comparisons with DPP4 and GLP1 RA were based on single pooled estimates demonstrating a reduction of 41% and 28%, respectively. SGLT2 inhibitors showed very heterogenic results regarding CV mortality, with ranges from a 5% increase to a 21% reduction compared to placebo, while all-cause mortality ranged from a 3% to 25% reduction. Compared to DPP4 inhibitors, SGLT2 inhibitors showed a more consistent benefit with a reduction in both cardiovascular and all-cause mortality, whereas compared to GLP1 RA, they showed a neutral pooled effect. Regarding MI and stroke, the systematic reviews and meta-analyses reported modest or neutral effects of SGLT2 inhibitors. More detailed information can be found in Table 3. The narrative reviews included in this review supported the results from the systematic reviews and meta-analyses, particularly regarding the reduction in MACE and HHF associated with the use of SGLT2 inhibitors.

Evidence from observational comparative effectiveness studies provided real-world estimates of the cardiovascular effectiveness of SGLT2 inhibitors across other active drug classes. Both GLP1 RA and SGLT2 inhibitors reduce the risks, but no significant difference in the quantitative measures is reported, with several studies showing non-significant differences in the reduction between the two classes. In contrast, between SGLT2 inhibitors and DPP4 inhibitors, SGLT2 inhibitors showed lower MACE events, with between 14 and 28%, consistent through the analyses. In addition, a reduction in hospitalisation for heart failure is consistently reported. Across comparators, SGLT2 inhibitors demonstrated promising results: vs. DPP4 inhibitors, 14% to 54% reduction, vs. GLP1 RA, up to 5% reduction, and vs. oGLDs, between 19% and 53%. SGLT2 inhibitors show substantial mortality benefits compared to DPP4 inhibitors, with cardiovascular mortality (CV) reduced by 15–63% and all-cause mortality reduced by 15–26%. Results for both cardiovascular and all-cause mortality varied substantially against GLP1 RA or oGLDs, showing wider and less consistent ranges from reduction to increase in the risk. SGLT2 inhibitors demonstrated a very modest to neutral effect on both myocardial infarction and stroke across different groups of comparators. Regarding the risk of stroke only compared to the DPP4 inhibitor, results show a consistent benefit with a reduction of between 14% and 15%.

**Table 3:** Summary of systematic reviews, meta-analyses, and narrative reviews evaluating the effectiveness of SGLT2 inhibitors in type 2 diabetes.

Study	Study Type	No. of Studies Included	Mean Age	Main Findings—Cardiovascular	Main Findings—Renal	SGLT2 Inhibitor	Comparator	Cardiovascular Outcome *	Effect Estimate (RR or HR, 95% CI)	Reanal Outcome *	Effect Estimate (RR or HR, 95% CI)
Drake et al., 2024 [19]	Systematic review and network meta-analysis of RCTs	84	58.7 y	Reduced HF hospitalisation and all-cause mortality; modest MACE reduction; neutral MI and stroke.	Significantly reduced composite kidney outcomes and progression of chronic kidney disease	SGLT2 inhibitors (class)	DPP4 inhibitors	MACE	RR 0.88 (0.80–0.97)	CKD progression	RR 0.62 (0.52–0.74)
							oGLDs (usual care)	MACE	RR 0.90 (0.83–0.98)	CKD progression	RR 0.66 (0.58–0.75)
								All-cause mortality	RR 0.86 (0.80–0.93)		
								CHF hospitalisations	RR 0.64 (0.54–0.77)		
								MI	RR 0.97 (0.85–1.12)		
								Stroke	RR 0.86 (0.77–0.95)		
							oGLDs (SU)	MACE	RR 0.57 (0.36–0.91)		
Wheeler et al, 2020 [23]	Narrative (non-systematic) review	NR	NR	Reduced CV death and hospitalisation for heart failure across CVOTs.	Slowed eGFR decline; reduced albuminuria; significantly reduced progression to ESKD and composite renal outcomes	Empagliflozin, Canagliflozin, Dapagliflozin, Ertugliflozin	Placebo or oGLDs (standard of care)	HHF	NR	Composite renal outcome	HR 0.54 (0.40–0.75)–0.81 (0.63–1.04)
								CV death			
Sinha et al., 2024 [25]	Systematic review and network meta-analysis	12 studies: 5 RCTs, 7 observational	mid-50 s to mid-60 s	Greater effectiveness for cardiovascular outcomes, with strongest and most consistent benefit for HF hospitalisation.	NR	Dapagliflozin	Placebo	MACE	HR 0.87 (0.75–1.01)	NR	NR
								HHF	HR 0.70 (0.59–0.84)		
								CV mortality	HR 0.79 (0.57–1.09)		
								MI	HR 0.89 (0.79–1.00)		
						Empagliflozin	Placebo	MACE	HR 0.86 (0.70–1.06)		
								HHF	HR 0.71 (0.59–0.86)		
								CV mortality	HR 0.84 (0.61–1.17)		
								MI	HR 0.87 (0.75–1.01)		
Singh et al., 2023 [14]	Systematic review	NR	NR	Consistent reduction in HF hospitalisation and CV death/HF composite across large CV outcome trials.	Significantly reduced composite kidney outcomes and progression of chronic kidney disease **	Dapagliflozin, Canagliflozin, Empagliflozin, Ertugliflozin, Remogliflozin	Metformin; placebo; standard of care	HFF	HR 0.47, 95% CI 0.41–0.54	Composite renal outcome	RR 0.67 (0.52–0.86)
										ESKD	RR 0.75(0.66–0.85)
										AKI	RR 0.75(0.66–0.85)
								CV death	NR	CKD progression	RR 0.55 (0.48–0.64)
Kalluri et al., 2021 [26]	Systematic review	22 studies (18 RCTs, 4 observational studies)	NR	Consistently reduced heart failure events (including hospitalisation), cardiovascular mortality, all-cause mortality, and nonfatal myocardial infarction across randomised and observational studies.	Slower kidney disease progression and reduced adverse renal outcomes **	Empagliflozin, Dapagliflozin, Canagliflozin, Luseogliflozin	Placebo; oGLDs	MACE	NR	NR	NR
								HHF			
								CV mortality			
								All-cause mortality			
								MI			
Caparrotta et al., 2021 [27]	Systematic review	37 observational studies	NR	Consistent reductions in hospitalisation for heart failure and modest reductions in major adverse cardiovascular events and all-cause mortality compared with other glucose-lowering therapies.	NR	Empagliflozin, Canagliflozine, Dapagliflozine	oGLDs	MACE	HR range: 0.78–0.94	AKI	HR Range 0.40–0.96
								HFF	HR range 0.48–0.79		
								All-cause mortality	HR range: 0.48–0.79		
Nyström, 2024 [31]	Narrative review		57–64 y	Consistent reductions in hospitalisation for heart failure, all-cause mortality, and modest reductions in MACE across large real-world cohorts.	Slower eGFR decline and significantly reduced progression to kidney failure and ESRD in real-world settings	Empagliflozin, Dapagliflozin, Canagliflozin, Ertugliflozin	DPP4 inhibitors	MACE	HR range: 0.76 (0.69–0.84)–0.94 (0.84–1.06)	AKI	HR range: 0.47 (0.27–0.80)–0.59 (0.48–0.73)
								HHF	HR Range: 0.43 (0.37–0.51)–0.94 (0.77–1.15)		
								CV mortality	HR range: 0.60 (0.54–0.96)–0.84 (0.65–1.08)		
								All-cause mortality	HR range: 0.55 (0.48–0.63)–0.80 (0.69–0.92)		
								MI	HR range: 0.70 (0.61–0.81)–1.02 (0.88–1.18)		
								Stroke	HR range: 0.69 (0.61–0.78)–0.94 (0.77–1.15)		
							GLP-1 RA	MACE	HR range: 0.90 (0.82–0.99)–1.03 (0.79–1.35)	AKI	HR 0.69 (0.45–1.05)
								HHF	HR range: 0.50 (0.44–0.56)–0.61 (0.47–0.78)	ESKD	HR 0.75 (0.60–0.94)
								CV mortality	HR 0.81 (0.67–0.97)	Composite renal outcome	HR 0.42 (0.34–0.53)
								All-cause mortality	HR range: 0.77 (0.32–1.85)–0.99 (0.78–0.99)		
								MI	HR range: 0.95 (0.86–1.05)–1.05 (0.79–1.35)		
								Stroke	HR range: 0.85 (0.66–1.10)–1.22 (0.95–1.22)		
							Placebo	MACE	HR range: 0.86–0.93	AKI	HR0.79 (0.72–0.88)
								HHF	HR range: 0.65 (0.50–0.85)–0.73 (0.61–0.88)	Composite renal outcome	HR range: 0.60 (0.47–0.77)–0.76 (0.67–0.87)
								CV mortality	HR range: 0.62 (0.49–0.77)–0.98 (0.82–1.17)		
								All-cause mortality	HR range: 0.68 (0.57–0.82)–0.93 (0.82–1.04)		
								MI	HR range: 0.85 (0.69–1.05)–0.91 (0.84–0.99)		
								Stroke	HR range: 0.90 (0.70–1.15)–1.24 (0.92–1.67)		
							oGLDs	MACE	HR range: 0.50 (0.45–0.55)–1.05 (0.94–1.23)	AKI	HR range 0.59 (0.46–0.74)–0.94 (0.60–1.47)
								HHF	HR range: 0.39 (0.26–0.60)–0.70 (0.61–0.81)		
								CV mortality	HR range: 0.53 (0.40–0.71)–1.34 (0.47–3.87)		
								All-cause mortality	HR range: 0.49 (0.41–0.57)–1.34 (0.47–3.87)		
								MI	HR range: 0.72 (0.63–0.82)–1.07 (0.83–1.37)		
								Stroke	HR range: 0.61 (0.50–0.73)–1.05 (0.90–1.23)		
Maremmani et al., 2025 [32]	Systematic review and meta-analysis	9 studies (5 RCTs, 4 PSM observational)	62.9 ± 10.5 y	Reduced all-cause mortality and hospitalisation for heart failure after myocardial infarction, with consistent benefit for early and delayed initiation.	NR	Dapagliflozin, Empagliflozin, Canagliflozin, Ipragliflozin	Placebo or no SGLT2i	HHF	HR 0.75 (0.67–0.83)	NR	NR
								All-cause mortality	HR 0.79 (0.68–0.91)		
Ahmed et al., 2024 [33]	Systematic review and meta-analysis	5 RCTs	61.8 y	Significantly reduced hospitalisation for heart failure after acute MI, with no significant effect on mortality outcomes.	NR	Empagliflozin, Dapagliflozin	Placebo	HHF	RR 0.76 (0.61–0.88)	NR	NR
								All-cause mortality	RR 1.05 (0.78–1.41)		
								CV mortality	RR 1.05 (0.78–1.41)		
Cao et al., 2022 [35]	Systematic review and network meta-analysis	16 RCTs	NR	Significant reductions in MACE, cardiovascular death, and hospitalisation for heart failure compared with placebo.	Significantly reduced composite renal outcomes and progression of diabetic kidney disease and ranked highest among glucose-lowering therapies for renal protection	Empagliflozin, Canagliflozin, Dapagliflozin, Ertugliflozin, Sotagliflozin	Placebo	MACE	HR 0.83 (0.76–0.91)	Composite renal outcome	HR 0.64 (0.57, 0.71)
								HHF	HR 0.86 (0.76–0.97)	CKD progression	HR 0.70 (0.63–0.77)
								CV death	HR 0.64 (0.56–0.73)		
							GLP-1 RA	MACE	HR 0.95 (0.82, 1.10)	Composite renal outcome	HR 0.74 (0.62–0.88)
								HHF	HR 0.72 (0.56, 0.92)		
								CV death	HR 0.97 (0.78, 1.22)		
							DPP-4 inhibitors	MACE	HR 0.81 (0.71, 0.92)	Composite renal outcome	HR 0.64 (0.52–0.79)
								HHF	HR 0.59 (0.49, 0.71)		
								CV death	HR 0.85 (0.71, 1.02)		
Bonnet et al., 2024 [36]	Narrative review	NR	NR	Consistent reductions in hospitalisation for heart failure, all-cause mortality, and modest reductions in MACE across real-world cohorts, including patients with CKD.	Slower eGFR decline, reduced risk of kidney failure and ESRD, and lower rates of acute kidney injury across CKD stages	Empagliflozin, Dapagliflozin, Canagliflozin	DPP-4 inhibitors GLP-1 RA oGLDs	MACE	NR	ESKD	HR range: 0.37 (0.25–0.55)–0.62 (0.51–0.75)
							GLP-1 RA	HHF			
										ESKD	HR: 0.75 (0.60–0.94)
										AKI	HR 0.85 (0.79–0.92)
							oGLDs			ESKD	HR range: 0.26 (0.11–0.61)–0.48 (0.33–0.71)
Martínez-Vizcaíno et al., 2021 [39]	Systematic review and network meta-analysis	Systematic review and network meta-analysis	63–64 y	Reduced all-cause mortality, MACE, and hospitalisation for heart failure in patients with T2DM at high CV risk; empagliflozin showed the highest probability of benefit, while ertugliflozin appeared less effective.	Significantly reduced composite renal outcomes	Empagliflozin, Canagliflozin, Dapagliflozin, Ertugliflozin	Placebo	MACE	HR 0.91 (0.85–0.97)	Composite renal outcome	HR 0.61 (0.50–0.74)
								HHF	HR 0.70 (0.62–0.78)		
								All-cause mortality	HR 0.85 (0.75–0.97)		

NT- not relevant; * Definitions of MACE and composite cardiovascular and composite renal outcomes varied by study and are reported as defined by the original authors. ** Renal outcomes were reported descriptively across included trials without pooled or quantitative effect estimates.

### 3.5. Renal Outcomes

A renoprotective role of SGLT2 inhibitors is fully supported throughout the reviewed studies [17,18,19,20,22,23,24,25,27,28,30,31,34,35,36,39].

Across systematic reviews and meta-analyses, SGLT2 inhibitors consistently demonstrated favourable renal outcomes compared to placebo. SGLT2 inhibitors reduce the relative risk of CKD progression by approximately 30% versus placebo and composite renal outcomes by between 36% and 39%. In addition, systematic reviews reported a lower risk of acute kidney disease (AKI) with approximately 25%. Compared with other active glucose-lowering therapies, SGLT2 inhibitors showed similar benefits, with detailed comparator-specific results summarised in Table 3. For end-stage kidney disease (ESKD), evidence was more limited and heterogeneous, with a protective effect reported in a single systematic review, whereas narrative reviews described substantial ESKD risk reduction across different comparators. Narrative reviews also supported the renoprotective profile of SGLT2 inhibitors.

Across the observational studies, SGLT2 inhibitors compared to other classes were associated with reduced risk of AKI, although these findings are reported in a limited number of studies. Evidence shows that risk reduction ranged from approximately 23% to 24% vs. GLP1 RA, 36% vs. DPP4 inhibitors, and 6% vs. oGLDs. Benefits were more significant in patients with pre-existing renal impairment, indicating potential advantages for high-risk patients. Findings about CKD indicate that SGLT2 inhibitors slow down the CKD progression, with ranges differing between the different classes. For end-stage kidney disease, a single observational study reported risk reduction, but with approximately 53% compared to oGLDs [34]. Comparisons with DPP4 inhibitors and GLP1 RA were not reported for ESKD in the observational studies included in this review. The risk reduction in composite renal outcomes and renal events associated with SGLT2 inhibitors was consistently observed across several observational studies, confirming their nephroprotective properties.

## 4. Discussion

Our systematic integrative review evaluated the effectiveness of SGLT2 inhibitors in glycaemic control, cardiovascular event reduction, as well as renal protective benefits among patients with type 2 diabetes mellitus based on studies performed in real-life clinical practice or systematising such. The method we used to collect data between January 2020 and January 2025 allowed us to gather a very large pool of information, which included both results from clinical trials as well as real-world evidence for the past five years. The synthesised evidence consolidates the existing body of data from the past years that SGLT2 inhibitors provide far greater benefits than just glucose lowering in the face of cardiovascular and renal benefits. That gives them one of the leading roles in diabetes therapy, especially for high-risk patients. Additional references not included in the formal systematic review were also cited to compare our findings with the recently published literature outside the predefined scope of this review and to further broaden the contextual interpretation.

Regarding glycaemic control, SGLT2 inhibitors show a modest but clinically significant reduction in HbA1c levels. Studies, which did not fall under the scope of our review, also show similar results. In an article studying the efficacy and safety of 11 different SGLT2 inhibitors, compared to placebo, SGLT2 inhibitors reduce the HbA1c levels with a mean difference of −0.45 to −0.80% [41]. Another study shows a dose–response relationship for the HbA1c reduction with an estimated maximum reduction of around 0.80% [42]. Still, improvement of the glucose levels and HbA1c varies substantially between studies, probably due to differences in baseline, variability in the patients’ therapy and other factors. SGLT2 inhibitors show better results in the parameters related to glucose level compared to DPP4 inhibitors, whereas GLP1 RA are often superior and succeed in showing better results. A study containing 61 RCTs showed that GLP1 RA are superior to SGLT2 inhibitors in HbA1c reduction with a mean difference of -0.39%, but also stated that GLP1 RA has a higher risk of adverse events [43]. Another article, which compared DPP4 inhibitors and SGLT2 inhibitors, demonstrated that at ≤26 weeks, there is no significant difference in HbA1c levels, but at ≥52 weeks, there is a statistically significant reduction in favour of the SGLT2 inhibitors with around -0.11%. The effects of SGLT2 inhibitors on glycaemic control remain sufficient for use as adjunctive therapy, especially when the therapeutic goals include benefits beyond glucose control.

The cardiovascular benefits of SGLT2 inhibitor use were strongly supported across the review literature. Compared to placebo and DPP4 inhibitors, consistent reductions in MACE, cardiovascular mortality and all-cause mortality were observed throughout the review. Against GLP1 RA, a class that also provides good cardiovascular benefits, no superiority was found regarding the mentioned events. SGLT2 inhibitors showed excellent results in the reduction of heart failure hospitalisation superior to all other comparators. Real-world evidence demonstrates even better results compared to the data from the RCTs in the reviews. The real-world evidence may show a stronger effect due to different reasons, such as a broader patient population or a longer follow-up period. However, observational studies may be suffering from selection bias as patients are not randomised. This should also be considered when comparing the results. A recently published study among 37,231 patients with type 2 diabetes showed a statistically significant reduction in both heart failure incidence and HHF rate among patients treated with SGLT2 inhibitors compared with those receiving GLP1 RA or neither therapy [44]. The effects on myocardial infarction and stroke were modest to neutral across most comparators. That suggests that the cardioprotective mechanism of SGLT2 inhibitors may primarily involve hemodynamic and renal pathways, rather than direct atherothrombotic benefits. Newer studies demonstrate that SGLT2 inhibitors show a reduction in stroke and in MI with 54% and 51%, respectively, compared to non-users [45]. Another recent study showed that treatment with SGLT2 inhibitors in patients with type 2 diabetes after a MI was associated with a lower rate of cardiovascular events, with 30% lower hazard of the composite outcome [46]. The apparent differences between current findings reflect the variations in patient population, baseline cardiovascular risk, as well as the study design and the follow-up period. Findings suggest that such benefits are more evident in specific high-risk populations on longer-term treatment. This is consistent with evidence pooled in the meta-analysis by Zelniker et al., where a reduction in MACE was established only in patients with atherosclerotic cardiovascular disease [47].

Consistent evidence of nephroprotective effects was demonstrated in this review. Reduction in acute kidney injury, slower progression of chronic kidney disease and lower incidence of end-stage kidney disease were reported across almost all of the included analyses. SGLT2 inhibitors were shown to be superior to all of the comparators, with the biggest difference against DPP4 inhibitors. Further recent evidence shows a study with 6946 patients in the course of 12 years reported that SGLT2 inhibitors are associated with a 41% lower risk of composite major adverse kidney events, as well as with a 48% less frequent progression to CKD stage 5 [47]. This identified consistency of renal benefits observed across different study designs and populations suggests that there is probably a class-related mechanism of action supporting the renoprotecting functions rather than specific study conditions [48]. Meta-analytic evidence, provided by Neuen et al., further supports a consistent reduction in major kidney outcomes across different trials and baseline levels of kidney function and albuminuria [49].

The heterogeneity of the studies, which are included in our review, should be considered when interpreting the findings. Our review is combining data from systematic reviews and meta-analyses, which contain data from RCTs as well as real-world observational comparative effectiveness studies. Observational studies often have a broader patient population, potentially with more co-morbidities. This can explain the differences in outcome estimates. Another reason can be the baseline patient characteristics. They vary between studies—factors like the duration of the diabetes, the patient’s risk profile, as well as the concomitant medication that is used, can severely contribute to the variability of the reported benefits. It is observed that studies including higher-risk populations demonstrate better results. Differences between comparator treatments can also influence relative effectiveness estimates and can partly explain inconsistencies between the analyses. Variation in the duration of the follow-up period, as well as the outcome definition itself across the different studies, affects the consistency of the results of the cardiovascular and renal outcomes. Such variability is consistent with established methodological principles. Differences in design, study population, comparator, and follow-up duration may contribute to heterogeneity across studies [50]. Taking all those described factors together, the overall direction of the benefits of the SGLT2 inhibitors looks consistent, but the magnitude of the effects strongly depends on the study context.

SGLT2 inhibitors provide a high multidimensional benefit profile beyond glucose-lowering alone. Their cardiorenal protective role is clearly shown and consistently highlighted in heart failure hospitalisation and renal disease progression. This perspective highly supports their use in patients with type 2 diabetes and increased cardiovascular ot renal risk.

### Strengths and Limitations

The current systematic integrative review has significant strengths as it thoroughly evaluates the impact of SGLT2 inhibitors on glucose levels, cardiovascular events, and renal function in patients with type 2 diabetes. The review provides a comprehensive perspective on the clinical benefits related to SGLT2 inhibitors by combining reviews containing randomised controlled trials and observational studies. Also including comparative effectiveness studies in the analyses allowed us to compare the effectiveness of the SGLT2 inhibitors against other glucose-lowering agents. This systematic review of reviews provides evidence to inform both clinical practice and future research. A key strength is the integration of multiple levels of evidence within a single structured framework. This allowed direct comparison between data from a controlled environment and real-world data.

Our approach allowed us to explore a more comprehensive understanding of the effectiveness of the SGLT2 inhibitors across different clinical settings as well as a diverse patient population. We focused on more than one endpoint, which provided us with a broader perspective that reflects current clinical decision-making, where treatment choices are often guided by the additional benefits of the therapy. Including mixed levels of evidence allowed us to draw parallels between the findings in a controlled environment and the real-world clinical practice. Our narrative synthesis was able to identify consistent patterns across outcomes and studies despite the heterogeneity, while acknowledging the areas of variability.

One of the main limitations that can be acknowledged is the heterogeneity in study designs, patient populations, and treatment durations. Variability across studies may be explained by differences in baseline characteristics and adherence to treatment. Another related limitation coming from the heterogeneity is the lack of quantitative synthesis. Heterogeneity does not allow graphical summaries like forest plots, which can enhance readability and facilitate the interpretation of complex findings, but may be misleading due to the narrative synthesis approach and the heterogeneity of the included studies. The narrative synthesis allows comparison of the overall trends, but the absence of meta-analysis may reduce the precision and robustness of the conclusions. As a result, the magnitude of the observed effect should be interpreted cautiously. In addition, the inclusion of mixed levels of evidence may introduce overlap of primary studies across the included literature. Although we did not perform quantitative synthesis, this may still influence the overall interpretation of the results and should be considered a limitation for our study. Despite this limitation and the potential risk of overlap of certain primary studies across included reviews, it is unlikely that such overlap substantially influenced the direction of the observed evidence. This interpretation is supported by the consistency of findings across different study designs and methodologies, with similar trends demonstrated throughout the studies.

Furthermore, information regarding adverse events and safety concerns was not the primary point of this research—thus, it remained limited. Conclusive evidence on the long-term benefits and risks of SGLT2 inhibitors needs to be compiled in future research focused on conducting well-designed trials with standardised methodology.

Another limitation is potential publication bias due to the fact that the search was conducted exclusively on PubMed. The exclusion of other databases may have resulted in missing relevant publications and introduced a risk of selection bias. Future reviews could benefit from multi-database search strategies to ensure more comprehensive evidence identification.

## 5. Conclusions

The findings from this systematic integrative review indicate that SGLT2 inhibitors have been proven to be effective in lowering HbA1c, reducing cardiovascular risk, and preserving renal function. However, the magnitude of benefit varies across different outcomes. More consistent effects are observed in heart failure hospitalisation and renal endpoint, while effects on outcomes such as myocardial infarction and stroke were generally more modest. Although their glucose-lowering effect is moderate, their pronounced cardiovascular and renal benefits compared to placebo and DPP4 inhibitors make them a preferred alternative for patients at increased cardiovascular or renal risk. Our findings highlight the multidimensional cardiorenal benefits beyond just glycaemic control. While some uncertainties remain about long-term safety and effects, the findings support the growing use of SGLT2 inhibitors as a central part of diabetes care.

## 6. Future Directions

Systematic reviews provide structured, systematised information about the focus and results of treatment effects studies. Future direction to enlarge findings in the area could include reviews focusing on multiple outcomes with new SGLT inhibitors; defining the time when the treatment with SGLT inhibitors should be initiated to prevent kidney damage.

## Data Availability

No new data were created or analyzed in this study. Data sharing is not applicable to this article.

## Supplementary Materials

The following supporting information can be downloaded at: https://www.mdpi.com/article/10.3390/pharmacy14020047/s1, File S1: PRISMA 2020 checklist; Table S1: Excluded studies.
